# Amiodarone-Induced Leukocytoclastic Vasculitis in a Decompensated Heart Failure Patient: A Case Report

**DOI:** 10.7759/cureus.51817

**Published:** 2024-01-07

**Authors:** Ummul Asfeen, Rohan Raj, Chinedu J Ezeafulukwe, Omar A Hassan, Deepa Treesa Francis, Sukhmeet S Dhillon, Aadil Khan

**Affiliations:** 1 Internal Medicine, New York Medical College, Saint Michael’s Medical Center, Newark, USA; 2 Internal Medicine, Nalanda Medical College and Hospital, Patna, IND; 3 Internal Medicine, Windsor University School of Medicine, Cayon, KNA; 4 General Practice, Ondokuz Mayis University, Samsun, TUR; 5 Internal Medicine, Baba Farid University of Health Sciences, Patiala, IND; 6 Trauma Surgery, OSF Saint Francis Medical Center, University of Illinois Chicago, Peoria, USA; 7 Cardiology, University of Illinois Chicago, Illinois, USA; 8 Internal Medicine, Lala Lajpat Rai Hospital, Kanpur, IND

**Keywords:** small-vessel vasculitis, leukocytoclastic vasculitis, decompensated heart failure, leukocytoclastic, vasculitis

## Abstract

Leukocytoclastic vasculitis (LCV) is a small-vessel vasculitis characterized by inflammation and damage to the walls of small blood vessels. It typically presents with palpable purpura and can be associated with various systemic conditions. Although its etiology is diverse, LCV has been associated with systemic diseases, infections, medications, and autoimmune disorders. Here, we present a case of LCV in a patient with decompensated heart failure. A 58-year-old man presented with progressively deteriorating swelling in both his lower limbs and scrotum, a persistent dry cough associated with minor ulcerative lesions on his shins, and a patchy rash with pustules and flat reddish spots. He was hospitalized three days prior due to atrial fibrillation and rapid ventricular rate, for which he was commenced on amiodarone. This rash persisted for three days, yet he denied experiencing any discomfort or itchiness along with it. Based on his clinical picture, laboratory evaluations, and imaging findings, he was diagnosed with LCV induced by amiodarone.

## Introduction

Leukocytoclastic vasculitis (LCV) is a small-vessel vasculitis characterized by inflammation of postcapillary venules, resulting in the deposition of immune complexes and subsequent damage to blood vessel walls. The emergence of small-vessel vasculitis is linked to conditions such as antineutrophil cytoplasmic antibody (ANCA)-associated vasculitis (e.g., microscopic polyangiitis, granulomatosis with polyangiitis), Behçet’s disease, and Cogan’s syndrome, and immune complex-related vasculitis is observed in conditions such as rheumatoid arthritis, lupus, and beyond [1,2]. Multiple other triggers can manifest, including infections, medications, autoimmune disorders, and malignancies, with up to 50% of cases having an unclear cause [3]. Amiodarone-induced LCV is rare and not widely reported [4,5]. While LCV is a relatively uncommon clinical entity, its association with certain medications, including amiodarone, has been documented in the medical literature. Amiodarone, a potent antiarrhythmic medication widely used in the management of cardiac arrhythmias, has been reported to cause various dermatologic adverse reactions, including LCV.

## Case presentation

A 58-year-old male was admitted due to progressively worsening bilateral lower extremity and scrotal edema associated with severe dyspnea, strongly suggestive of a heart failure exacerbation. The persistent rash, tender upon palpation, appeared prominently on the trunk and extremities. He had no history of prior skin disorders, recent fever, or chills. Additionally, he observed small ulcerating lesions on his shins and experienced a patchy pustular and maculopapular rash lasting for three days. He initially denied experiencing any pain or pruritus with this rash.

He was hospitalized two weeks ago due to atrial fibrillation and rapid ventricular rate, for which he was commenced on an amiodarone oral loading dose of 1,200 mg once daily. He was discharged in sinus rhythm on amiodarone, metoprolol 50 mg twice a day, and rivaroxaban 20 mg once a day. His past medical history revealed uncontrolled hypertension; however, he was not on any prescribed medications currently.

**Figure 1:**
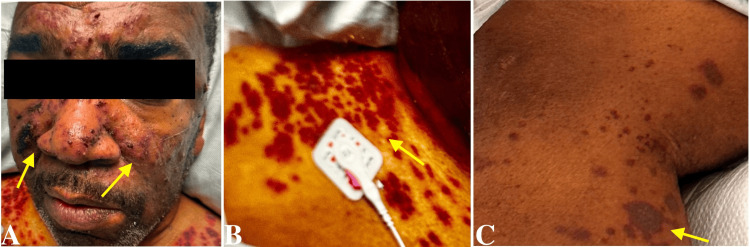
Scattered flaccid vesicles overlying violaceous plaques with superficial crusting across the nose, bilateral cheeks (A), chest (B), back, and lower extremities (C).

On examination, he had a heart rate of 120 beats/minute, blood pressure of 214/150 mmHg, and a respiratory rate of 25 breaths/minute. Oxygen saturation was at 95%, with the patient on a 4 L nasal cannula. A thorough physical examination revealed marked bilateral lower extremity pitting edema, gross scrotal swelling, edematous hands, and notable skin manifestations. These skin findings included scattered flaccid vesicles overlying violaceous plaques with superficial crusting across the nose, bilateral cheeks, chest, back, and lower extremities. A distinct hyperpigmented plaque was observed over bilateral inguinal folds, and scattered petechiae were present on the palmar surfaces of both hands. A patchy maculopapular rash extended across the torso and upper abdomen (Figure 1).

**Figure 2:**
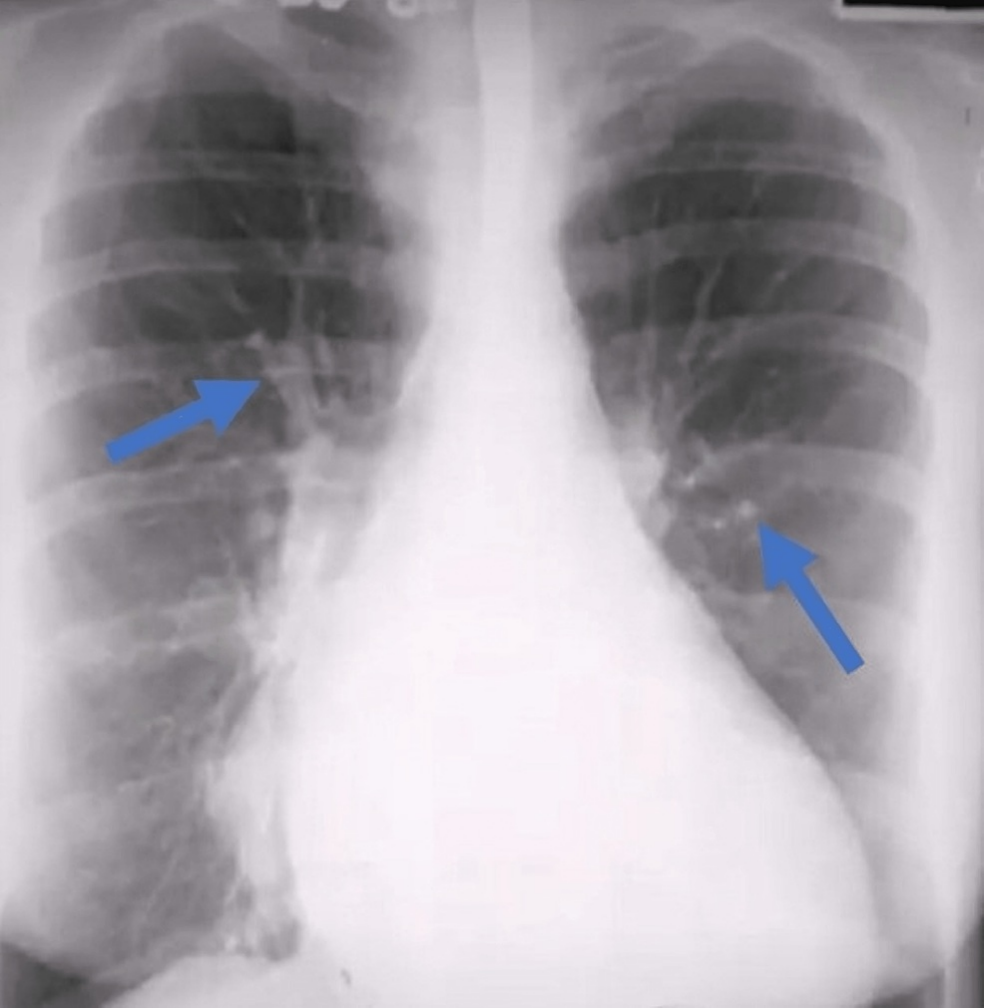
Chest X-ray revealing cardiomegaly, vascular redistribution, and interstitial edema.

**Figure 3:**
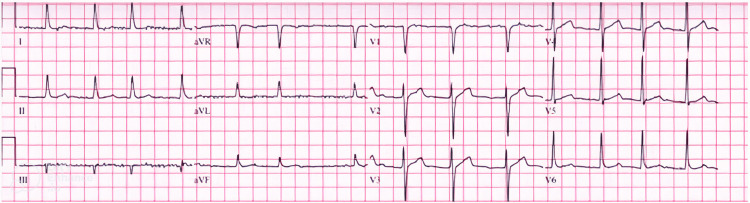
Electrocardiogram revealing atrial fibrillation with rapid ventricular response.

Initial results of serum and biochemical parameters are shown in Table 1. Blood cultures revealed no growth and urine analysis unveiled substantial proteinuria and hematuria. A chest X-ray was performed, which showed cardiomegaly, and an electrocardiogram was significant for atrial fibrillation (Figures 2, 3). The echocardiographic evaluation showed severe left ventricular hypertrophy, with an ejection fraction of 50-55%, suggesting diastolic dysfunction.

**Table 1:** Hematological and biochemical parameters.

Lab test	Result	Normal range
Troponin	120 ng/dL	<0.03 ng/mL
Hemoglobin	10.1 g/dL	13.5–17.5 g/dL
Creatinine	1.71 mg/dL	0.5–1.2 mg/dL
B-type natriuretic peptide	1152 pg/mL	<100 pg/mL
Urinalysis (protein)	300 mg/dL	<30 mg/dL
Urinalysis (blood)	3+	Negative
Alanine aminotransferase	58 U/L	7–56 U/L
Aspartate aminotransferase	73 U/L	8–38 U/L
Alkaline phosphatase	87 U/L	45–115 U/L
Total bilirubin	1.3 mg/dL	0.1–1.2 mg/dL
Erythrocyte sedimentation rate	50 mm/hour	<15 mm/hour
C-reactive protein	3.5 mg/dL	<1 mg/dL
Creatine kinase	966 U/L	26–308 U/L
Creatine kinase MB	10.6 ng/mL	0.6–6.3 ng/mL

He was managed with intravenous furosemide, aspirin-loading dose, and nitroglycerin. Amiodarone was discontinued, and atrial fibrillation resolved spontaneously without needing specific intervention. Throughout treatment, his systolic blood pressure exhibited fluctuations, and hydralazine was administered to address hypertensive episodes, resulting in a notable decrease in swelling and an overall improvement in the patient’s condition.

Given the complex presentation, a dermatology consultation was sought to assess the diffuse rash. The possibility of vasculitis versus staphylococcal scalded skin syndrome was considered. Nikolsky’s sign was absent. He underwent a biopsy of the skin lesion, and direct immunofluorescence revealed the deposition of immunoglobulins (Ig) G, IgA, C3, and fibrinogen in blood vessels and peri-eccrine basement membrane in the reticular dermis and diffuse stromal background deposition. IgM was negative. A final diagnosis of LCV was made due to amiodarone after ruling out all other possible causes. After discontinuing the medication, the persistent lesions vanished completely after being present for over 15 weeks. The patient responded positively to prednisolone, cyclophosphamide, and fluids. His condition improved satisfactorily. He was discharged with regular follow-ups, and there was no reappearance during a follow-up period of more than six months.

## Discussion

LCV exhibits a diverse range of triggers, encompassing infections, such as hepatitis B and *Streptococcus*, certain medications, including penicillin and antihypertensives, as well as various environmental and allergic factors [6,7]. Additionally, systemic conditions such as systemic lupus erythematosus, inflammatory bowel disease, and genetic predispositions have been associated with LCV development, with ongoing investigations into specific genetic variations [8-10].

Notably, there is a complex interplay between LCV and other medical conditions. Case reports have highlighted intriguing connections between LCV and various comorbidities or triggers. For instance, there have been cases where LCV flares were precipitated by factors such as influenza vaccination, certain infections such as *Mycoplasma*, and medications such as vancomycin or warfarin [11-18].

In particular, there is a growing understanding of LCV’s association with cardiac conditions. Instances such as LCV occurring alongside ischemic heart disease, heart transplant recipients undergoing immunotherapy, or cases within the context of heart failure reveal an intricate relationship between vasculitis and cardiac health [13,14,17,19]. Furthermore, systemic vasculitis in patients with conditions such as Sjögren’s syndrome can present with cardiomyopathy, emphasizing the need for recognizing vasculitis as a potential cause in such scenarios [20]. Table 2 presents a concise overview of each study’s primary focus and findings, enabling quick comprehension and comparison of the diverse associations between LCV and various comorbidities or triggers.

**Table 2:** Case studies of LCV and associated comorbidities/triggers. ANCA: antineutrophil cytoplasmic antibody; LCV: leukocytoclastic vasculitis

Study	Key findings
Ulm et al. [13]	LCV case with a history of ischemic heart disease exacerbated by an influenza vaccine, leading to acute renal failure requiring hemodialysis
Ippoliti et al. [14]	LCV occurrence post-heart transplant alongside immunotherapy, indicating a potential link between immunosuppression and vasculitis
Rao et al. [15]	LCV manifestation following Mycoplasma-induced upper respiratory tract superinfection, characterized by conspicuous skin lesions
Pingili et al. [16]	Diffuse LCV and acute renal failure observed after intravenous vancomycin administration, highlighting a medication-triggered LCV case
Elantably et al. [17]	LCV diagnosis subsequent to warfarin prescription for rheumatic heart disease, exhibiting classic manifestations of LCV
Missoum et al. [18]	Vaccine-induced LCV linked to the inactivated COVID-19 vaccine, diagnosed due to lack of alternative etiology post-vaccination
Orlando et al. [19]	LCV coexisting with heart failure showing reduced ejection fraction due to ANCA-negative vasculitides, emphasizing the cardiac implications of vasculitis
Golan et al. [20]	Systemic vasculitis in a patient with Sjögren’s syndrome, leading to exertional dyspnea and orthopnea, showing reversibility post-immunosuppressant therapy and improved ejection fraction

Among medications, amiodarone has been implicated in vasculitis, specifically LCV, although the exact mechanisms remain less understood [3-5]. Current literature suggests the potential involvement of immune complexes formed by amiodarone or its metabolites, triggering an inflammatory response within blood vessel walls, thereby leading to vasculitis [3,4].

Our patient’s complex presentation involves several potential triggers and factors contributing to his condition. The recent initiation of amiodarone aligns with the onset of symptoms, suggesting its role in triggering adverse effects such as vasculitis. Additionally, the patient’s underlying uncontrolled hypertension, coupled with cardiovascular issues such as atrial fibrillation, severe left ventricular hypertrophy, and diastolic dysfunction, may have predisposed him to heart failure exacerbation. The combination of multiple medications (amiodarone, metoprolol, and rivaroxaban) could have interacted, potentially influencing the severity or manifestation of adverse reactions [21]. Individual susceptibility, cumulative effects of medications, possible drug interactions, and an immune-mediated response to amiodarone might collectively contribute to the observed side effects, emphasizing the likelihood of amiodarone as the primary trigger for the vasculitic reaction observed in this case.

The multifaceted triggers and associations of LCV underscore the complexity of its manifestations and underline the necessity for clinicians to remain vigilant in identifying potential triggers, especially when treating patients with medications known to have vasculitis-related implications such as amiodarone. Further research is crucial to elucidate the underlying mechanisms and strengthen our understanding of these intricate relationships.

## Conclusions

Amiodarone-induced LCV is a rare adverse reaction associated with the use of this antiarrhythmic medication. Although the pathophysiology of amiodarone-induced LCV remains unclear, it is thought to involve immune complex deposition in blood vessel walls, leading to inflammation and tissue damage. Clinicians should be vigilant for dermatologic manifestations in patients receiving amiodarone, and a prompt evaluation is essential to confirm the diagnosis. Discontinuation of amiodarone is the mainstay of treatment, and systemic corticosteroids may be required in severe cases.
